# Toward a Taxonomy for Adaptive Data Visualization in Analytics Applications

**DOI:** 10.3389/frai.2020.00009

**Published:** 2020-03-20

**Authors:** Tristan Poetzsch, Panagiotis Germanakos, Lynn Huestegge

**Affiliations:** ^1^Department of Psychology, Julius-Maximilians-University Würzburg, Würzburg, Germany; ^2^User Experience ICD, Product Engineering, Intelligent Enterprise Group, SAP SE, Walldorf, Germany

**Keywords:** graph adaptivity, data visualization, user model, analytics, graph ergonomics, recommendation engine

## Abstract

Data analytics as a field is currently at a crucial point in its development, as a commoditization takes place in the context of increasing amounts of data, more user diversity, and automated analysis solutions, the latter potentially eliminating the need for expert analysts. A central hypothesis of the present paper is that data visualizations should be adapted to both the user and the context. This idea was initially addressed in Study 1, which demonstrated substantial interindividual variability among a group of experts when freely choosing an option to visualize data sets. To lay the theoretical groundwork for a systematic, taxonomic approach, a user model combining user traits, states, strategies, and actions was proposed and further evaluated empirically in Studies 2 and 3. The results implied that for adapting to user traits, statistical expertise is a relevant dimension that should be considered. Additionally, for adapting to user states different user intentions such as monitoring and analysis should be accounted for. These results were used to develop a taxonomy which adapts visualization recommendations to these (and other) factors. A preliminary attempt to validate the taxonomy in Study 4 tested its visualization recommendations with a group of experts. While the corresponding results were somewhat ambiguous overall, some aspects nevertheless supported the claim that a user-adaptive data visualization approach based on the principles outlined in the taxonomy can indeed be useful. While the present approach to user adaptivity is still in its infancy and should be extended (e.g., by testing more participants), the general approach appears to be very promising.

## Introduction

As the recent acquisition of analytics application provider Tableau by software giant Salesforce shows, the relevance of self-service data visualization software is rapidly increasing. Considering the associated commoditization, the user group for data analytics applications is not only becoming larger, but also more diverse, and so are personal backgrounds and levels of experience regarding data visualizations (Convertino and Echenique, 2017; Lennerholt et al., 2018). Although dealing with diversification is thus becoming more relevant, current research still focuses either on data-based recommendations for visualization generation (Viegas et al., 2007; Vartak et al., 2015; Wongsuphasawat et al., 2016) or on individual factors determining the processing of data visualizations (e.g., the data literacy concept) (Gal, 2002; Shah and Hoeffner, 2002; Roberts et al., 2013). However, these two important areas have not yet been sufficiently considered in conjunction, although the benefits of improving the accessibility of data through individualized visualizations may very tangibly contribute to achieving better business decisions. Hence, in this paper we explore the potential of the latter idea by enriching the current user models with specific human characteristics, and by following an experimental approach we propose a taxonomy for user adaptivity in data visualization as a foundation for further research.

In order to derive this taxonomic approach, we outlined three main research questions for this paper: Is there a need for a user-adaptive approach to data visualization? How should a taxonomy be structured in its user-adaptive approach? Can the usefulness of an adaptive approach be validated? Based on these research questions we structured the present paper, starting with an examination of previous works on data visualization. Based on this, we determined a user model consisting of user traits, states, and strategies as well as their respective operationalizations.

Following up on the theory, we conducted three studies to understand the need and the relevant factors for an adaptive approach to data visualization. *Study 1* explored how User Interface Design experts would visualize different data sets, thereby addressing the first research question regarding the need for an individualized approach. We hypothesized that recommendations by the experts would vary significantly, therefore supporting the need for an individualized approach. For the second research question on how a taxonomy should be structured, we conducted two follow-up studies based on the proposed user model. *Study 2* explored how user *traits* impact on the perception of different data visualization encodings, and hence laid the groundwork for adapting to traits. The associated hypothesis was that not all visualizations were suitable for every user. *Study 3* focused on understanding how user *states* can be operationalized as intents, and how these can differ from each other. Here the hypothesis was that different intents are characterized by different associated cognitive subtasks and should therefore significantly impact on visualization requirements. Both studies are necessary preconditions contributing to the design of an adaptive data visualization taxonomy.

Based on these insights, a general adaptive taxonomy of diagram choice, layout, and specific visualization design was derived. An important feature of this taxonomy is that it can handle multidimensional data and state- or trait-related user variables. The validation for the usefulness of the taxonomy as outlined in the third research question was addressed in *Study 4*, in which User Interface Design experts were asked to perform different tasks with visualizations suggested by the taxonomy and rated their experience afterwards. The findings indicated some potential for such a taxonomy, although there is still some work to be done before it may be applied in a consumer setting.

## Background and Previous Work

Data visualizations have been in use to present numerical information since the early twentieth century (Eells, 1926) and consequently spawned a research tradition that is still active (Cleveland and McGill, 1984; Shneiderman, 1992; Heer et al., 2010). Four larger research streams can be summarized under this umbrella. While at first the focus was on optimizing single visualizations (Gillian and Lewis, 1994), the requirement to display more complex information subsequently led to research into how multiple charts may be layouted next to each other in a “multi-view” perspective (Roberts, 2007). The rise of personal computers then enabled not only static displays, but also the possibilities to interact with data visualization, supported by the field of human factors (Stasko et al., 2008). Lastly, the research on automatic recommendations for data visualizations became increasingly relevant as more users without an academic degree in statistics or computer science gained access to self-service analytics solutions (Mackinlay, 1986; Stolte and Hanrahan, 2000; Wongsuphasawat et al., 2016). All these research streams combine challenges from various fields, including psychology, computer science, and human factors.

Before exploring these research streams in more detail, some terms need to be disambiguated first. A chart refers to a single visualization of a set of data, for example, a bar chart. Within such charts, an indicator refers to a graphical element that represents the value of a single data point associated with a variable, such as a single bar in a bar chart or a point in a scatterplot. The type of encoding/indicator refers to the kind of indicator in use, such as bars, points, or lines (Gillian and Lewis, 1994). Finally, another important distinction for the present purpose is that between adaptivity, personalization, and customization. While adaptivity refers to a system that automatically sets up functionality and a user interface to fit the user, personalization requires the user to actively set up the system in a way that fits him/her. In contrast, customization takes place when a third party sets up the system to fit the user (Germanakos, 2016). The following literature review on data visualization will refer to this basic terminology.

Research on data visualization was first concerned with the optimization of single chart visualizations, starting with Eells (1926). Corresponding research on data encoding effectiveness peaked in the 80s and 90s, when landmark studies like those by Cleveland and McGill (1984), who invented dot plots, or by Hollands and Spence (1992), who evaluated line charts vs. bar charts as the most effective means to communicate change in data (see also Huestegge and Philipp, 2011; Riechelmann and Huestegge, 2018), emerged. Scatterplots, on the other hand, were later considered an optimal choice for visualizing correlations (Harrison et al., 2014; Kay and Heer, 2016). Over the years, new visualization techniques such as tree maps were introduced (Shneiderman, 1992; Heer et al., 2010; Bostock et al., 2011). Besides encoding types, particular features of visualizations like color (Lewandowsky and Spence, 1989; Demiralp et al., 2014) or chart size were studied more closely. Regarding the latter, several studies emphasized that smaller charts (<17° of the visual field) were considered helpful in avoiding gaze shifts along with associated inaccuracies (Heer et al., 2009; Heer and Bostock, 2010; Strasburger et al., 2011; Orlov et al., 2016). Apart from data point reading accuracy, the size of a chart was also shown to influence perceptual strategies: While smaller graphs facilitated quick overall assessments and immediate responses to graphs, larger charts led to increased scrutiny during graph comprehension (Orlov et al., 2016).

As most data sets and real-world contexts are too complex to be displayed in a single chart, more research on multi-view visualizations emerged in the early 2000's. These flexible data visualization features also became more prominent in data analytics software. For example, pioneering applications such as snap-together visualization (North and Shneiderman, 2000) or Polaris (Stolte and Hanrahan, 2000) emerged. Historically, such multi-view visualizations originally consisted of dual-views of data (Roberts, 2007), comprising, for example, Overview + Detail, Focus + Context, or Difference Views, the latter involving two datasets that are laid out next to each other to facilitate comparison. Another line of research on more complex data sets focused on so-called “Small Multiples,” which depict the relationship of several variables relative to each other. This type of visualization was also combined with a master-slave approach, so that manipulating data in one view also affected visualizations in the other view (Roberts, 2007; Scherr, 2009; van den Elzen and van Wijk, 2013).

The development of these types of data visualization also stimulated research on interaction with corresponding graphs. Especially as interactive visualizations became more and more common at the end of the twentieth century, studies on using interactive graphs, which quickly became a standard in data analysis software, were on the rise. This was not surprising, since interactive visualizations offer many benefits for working with data, from providing context information to increasing attention (Stasko et al., 2008). With the onset of touch-based devices, an entirely new class of interactive data display solutions, with its own set of challenges, emerged: Especially with dashboards, main goals for designing applications for mobile devices comprised maximizing the size of each visualization, minimizing occlusion, keeping all visualizations in view, and reducing any need for end-user customization of views (Sadana and Stasko, 2016).

Finally, with increasing commercial interest in data visualization for large sets of data, automation of data visualization became an important issue. Self-organizing dashboards based on recommendation systems were developed as an answer to the disproportionally large amount of user time devoted to data handling (compared with the actual goal of conducting science; Howe and Cole, 2010). Automatic data visualization recommendations have come a long way (Mackinlay, 1986; Stolte and Hanrahan, 2000; Viegas et al., 2007; Vartak et al., 2015; Wongsuphasawat et al., 2016). Especially due to the commoditization of data analytics, recent recommendation engines such as Voyager 2 (Wongsuphasawat et al., 2017) are gaining increasing attention. The underlying criteria for these recommendation systems are best outlined along the axes data characteristics, intended task or insight, semantics and domain knowledge, visual ease of understanding as well as user preferences and competencies (Vartak et al., 2017). Taken together, research in this area already points toward further development of recommendation systems in the areas context sensitivity and, ultimately, user-adaptive data visualization.

## Structuring Individual Perception of Data Visualization Within a User Model

**Figure 1:**
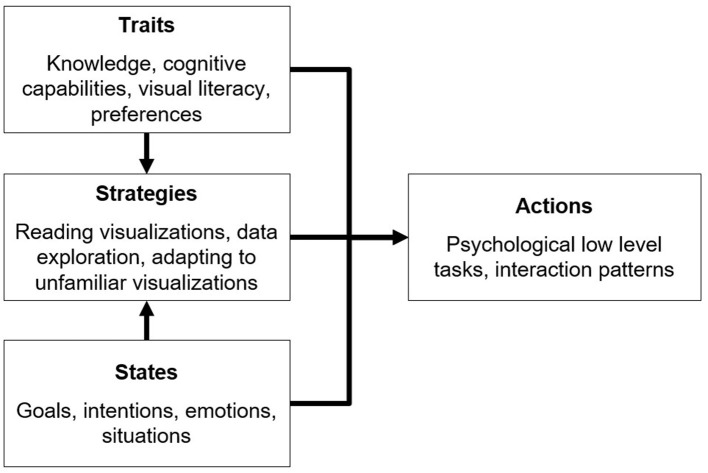
Modeling aspects of user-adaptive data visualization.

As mentioned above, adapting the display of data to the user is the next challenge in the field of data visualization, especially since information overload appears to become a major problem in business decisions (Moore, 2017), and therefore calls for user-specific approaches. However, structuring user-adaptive data visualization requires a user model in the first place (Germanakos, 2016). A useful basic distinction in this context is that between (relatively persistent) user traits and (more transient) situational states of the user (Kelava and Schermelleh-Engel, 2008). Based on various possible traits and the states, several strategies can be applied by users to deal with visualized data, as displayed in Figure 1.

Research on how individual traits can affect the perception of data visualization started around the 80's with the concept of graphical literacy, loosely defined as “*the ability to read and write (or draw) graphs*” (Fry, 1981, p. 383). Later, the concept was elaborated, and subdivided into the three skill levels “*reading the data*,” “*reading between the data*,” and “*reading beyond the data*” (Friel et al., 2001; Okan et al., 2012). Based on these theoretical considerations, fostering the development of graphical literacy became a focus of research (Gal, 2002; Shah and Hoeffner, 2002; Roberts et al., 2013). Furthermore, determining the cognitive variables underlying graphical literacy has also been of considerable interest. Commonly, perceptual speed, visual working memory and—to some extent—verbal working memory were discussed as potentially relevant factors in this regard, sometimes joined by locus of control (Velez et al., 2005; Conati and Maclaren, 2008; Toker et al., 2013; Lallé et al., 2015). Perceptual speed refers to the “*speed in comparing figures or symbols, scanning to find figures or symbols, or carrying out other very simple tasks involving visual perception*” (Conati and Maclaren, 2008, p. 202). Both verbal and visual working memory are part of the working memory architecture proposed by Baddeley (1992). Specifically, the visuospatial sketchpad comprises the ability to manipulate visual images, while the phonological loop stores and rehearses speech-based information. As perceptual speed and visual working memory have repeatedly been shown to be relevant traits regarding the perception of data visualization, we chose to include assessments of these constructs in our studies.

When outlining user states and user intentions in particular within the data analytics context, no widely accepted general model is currently present in the literature. It has been proposed that, on a higher processing level, one should distinguish visualization purposes into analysis, monitoring, planning, and communicating (Few, 2004). However, it should be noted that specifying the actual intention of a user and providing the appropriate information is certainly not a trivial challenge (Gotz and Wen, 2009; Conati et al., 2015; Oscar et al., 2017).

Previous research on strategies for reading data visualizations primarily focused on visual processing, reasoning with data points, and integrating context knowledge (Amar et al., 2005; Ratwani et al., 2008). Interindividual differences in strategies were assumed to be especially relevant in more unrestricted settings such as understanding unfamiliar visualizations (Lee et al., 2016), or in the context of designing visualizations (Grammel et al., 2010).

## Exploring the Need and the Relevant Factors for the Design of an Adaptive Taxonomic Approach

Although previous literature repeatedly recommended to put more research effort into studying user adaptivity in the context of data visualization (see above), we reasoned that it is mandatory to verify the need for an underlying taxonomic approach to user state/trait-based visualizations first. Therefore, we conducted *Study 1* with User Interface Design experts (*n* = 16) to explore the need for an adaptive taxonomic approach by letting them freely design visualizations for several data sets in order to see if the results would differ, thereby underlining the need for adaptivity.

Based on this, we conducted two follow-up studies to understand user traits and states in more detail based on a user model. *Study 2* (*n* = 45) had the goal of evaluating how individual traits and backgrounds affect the interpretation of different data visualization types. Therefore, the number of errors in the interpretation as well as interpretation speed were measured with 20 different visualizations. *Study 3* evaluated how different goal-states affected the decision which cognitive processing steps are taken in working with data visualization, and how—based on the taxonomy—visualizations should therefore adapt based on user goals. Two main goal-states (analysis vs. monitoring) were identified from theory, and analytics experts regularly working with numbers (*n* = 14) were questioned about their typical real-life tasks involving data. These tasks were split into their associated (low-level) cognitive processing steps and classified into one of the two goals. Based on the results of the conducted studies, a taxonomy was derived (see section Developing a User-Adaptive Visualization Taxonomy). All data sets from the studies are available online (link in section Supplementary Materials).

### Study 1—Exploring the Need for an Adaptive Approach

*Study 1* explored how User Interface Design experts would visualize different data sets. As we already anticipated the need for an adaptive approach based on an underlying taxonomy, we hypothesized that analytics expert participants would vary considerably in choosing a type of encoding for various given data sets.

#### Participants

Sixteen analytics experts (SAP employees, 7 female, 9 male) were tested and interviewed. The participants were chosen based on their experience within the context of data analytics. All participants had an academic background and were working for SAP for at least 6 months in the areas of UX or analytics. All participants also had considerable experience in working with data visualization (>1 year of professional experience). The age range was 27–48 years.

#### Stimuli

There were 16 data sets to be visualized by the participants. These data sets were of different complexity and constructed based on the combinations of four dichotomous factors: (a) single or multiple (three) numerical variables, (b) single or multiple (three) categorical variables, (c) data including or excluding time as a variable, and (d) data with 1:1 cardinality or a 1:n cardinality. In six of these data sets, geographical variables were included.

#### Procedure

After a brief introduction, the data sets were presented to the participants one after another on single sheets of paper by the experimenter. The participants were asked to sketch recommendations for respective visualizations on the same page as the data set. This was done in order to minimize a potential influence of software restrictions or software experience. The encoding recommendations were classified by the experimenter. The study lasted around 42 minutes (*SD* = 13).

#### Design and Data Analysis

The independent variables for designing the data sets were the number of categorical variables (1 or 3) in the dataset, the number of numerical variables (1 or 3) in the dataset, the cardinality of the data (1:1 or 1:n) and if a time variable was presented (yes or no). There was one data set for each combination of these independent variables. For each data set, we calculated the proportion of different (vs. same) visualizations designed across participants (e.g., a proportion of 100% would indicate that all participants came up with the same solution), which served as the dependent variable.

#### Results

The solutions proposed by the participants varied considerably, as indicated by a mean proportion of different visualizations of 51% (SD = 20.4). In some cases, all participants proposed different types of data visualizations. A one-sample *t*-test indicated that the mean significantly differed from 100%, *t*_(15)_ = 9.97, *p* < 0.0001. Therefore, the results generally support the assumption of substantial variability in individual visualization preferences. In addition, it was observed that most participants actively tried to reduce data complexity by plotting multiple, differently scaled numerical variables on the same axis (sometimes even stacking these differently scaled variables, resulting in misleading data representations). Another strategy to reduce complexity was the use of filters. Finally, we also observed that intra-individual consistency in chart choice (e.g., always using a geomap for geodata) tended to be low. As an additional exploratory analysis, we also conducted a multiple regression analysis using the independent variables (for designing the data sets) as predictors. This analysis resulted in a significant overall regression (R = 0.91, *p* < 0.001) with significant contributions of the predictors “number of categorical variables” (β = 0.66, *t* = 5.276, *p* < 0.001) and “number of numerical variables” (β = 0.58, *t* = 4.620, *p* = 0.001), while the remaining two predictors had no significant impact (β < 0.21, *t* < 1.7, *p* > 0.12). Specifically, an increase in the number of variables led to more diverse solutions.

#### Discussion

The variability present in the results of this exploratory study generally supports the call for user-adaptive data visualization. Participants suggested different visualizations for the same data sets, even though general user characteristics such as their academic background and field of work were relatively similar. In the context of the proposed high-level user model, the results therefore suggest that it may be worthwhile to study potential effects of more specific user traits and strategies on visualization selection and design to eventually optimize and support visualization decisions. In addition, as most participants had problems with dealing with the inherent complexity of the data, a taxonomic approach that not only takes user variables but also (multidimensional) data characteristics into account would clearly be desirable.

#### Limitations

There were several shortcomings in this exploratory study that need to be discussed. First, due to time restrictions eight of the 16 participants were not able to complete all tasks, and thus the results of the analyses should only be interpreted with great care. Second, the approach of this study lacked some degree of ecological validity, as participants were asked to choose visualizations without the help of a dedicated software. In a brief interview at the end of the study protocol, several participants commented that they would actually click through all available alternatives in a given software instead of actively developing a visualization concept. Third, all participants were employees of SAP and therefore almost certainly affected by the company's design language and typical visualization solutions, even though the heterogeneity of the results implied that this did clearly not result in similar outcomes among participants. However, one might suspect that the results might vary even more substantially if analysts or analytics UI experts from other companies were added to the sample.

### Study 2—Examining the Perception of Various Visualizations Considering User Traits

As *Study 1* suggested the need for a user-adaptive approach to data visualization, the next step was to derive more specific research questions based on the user model outlined above. Starting with user traits, *Study 2* aimed at becoming more specific about determining which traits may be relevant for the development of an adaptive taxonomy, especially regarding the selection of specific types of visual encoding. Based on results from previous literature (see above), we specifically focused on prior experience, visual literacy, and cognitive capacities. These factors were considered relevant for the participants' ability to understand and work with a wide variety of data visualizations. More specifically, we hoped that it is possible to classify individual data visualizations into those more suitable for experts or novices in order to take this issue into account within the taxonomy. This was done using a cluster analysis approach.

Consequently, the main hypothesis in this study was that some visualizations are more appropriate for participants with substantial prior experience, visual literacy, or cognitive capacities to adapt quickly to these visualizations. Prior experience was operationalized in terms of education, working in a data-driven job, and the degree of statistical knowledge. To measure graphical literacy, the *Subjective Graphical Literacy Scale* (SGL) (Galesic and Garcia-Retamero, 2011; Garcia-Retamero et al., 2016) was used. Cognitive capacities were tested by assessing perceptual speed and visual working memory. To measure perceptual speed, a *Sum to 10 test* (Ackerman and Beier, 2007) was used as it is also based on numerical (and not only visual) abilities. In this test, participants are presented with combinations of two numbers, and have to decide quickly if their sum is equal to 10 or not. To measure visual working memory, a *Visual Patterns Test* based on the Visual Patterns Test by Della Sala et al. (1999) was administered. In this test, participants are exposed to a black and white pattern grid and have to recognize this pattern from a selection of similar patterns after a brief distraction interval. A multiple regression analysis was used to test which of these several traits significantly predict graph comprehension abilities.

#### Participants

All participants (*N* = 45) were recruited via Social Media. Thirty-three participants were male (73%), 12 Participants were female (27%). Thirteen of the participants had a high school degree, 19 a bachelor's degree and 12 a master's degree, and one had finished an apprenticeship.

#### Stimuli

The following data visualization types were evaluated: Scatterplot, Area Chart, Stacked Bar Chart, Stacked Area Chart, Boxplot, Bullet Chart, Waterfall Chart, Bubble Chart, Heatmap, Treemap, Sunburst, Sankey Chart, Matrix Scatterplot, Trellis Bar Chart/Small Multiples, Sparklines and Horizon Charts (see Heer et al., 2010, for details on these visualization types).

#### Procedure

The experiment was web-based and therefore completed on the participants' own devices. The experiment was designed and conducted using the platform soscisurvey (www.soscisurvey.com). After a brief introduction to the study and its parts, participants were asked to report their highest educational degree. After that, they were asked if their job involved a lot of work with numbers and graphs on the scale “No”—“Sometimes”—“Yes.” Additionally, participants were asked to rate their familiarity with statistics and data interpretation on the following scale: “Not familiar at all”—“Somewhat familiar (e.g., familiar with averaging)”—“Familiar (e.g., familiar with correlation, variability measures, different types of distributions including normal distributions)—“Very familiar (e.g., familiar with factor analysis, cluster analysis, ANOVA).” After this, participants completed the SGL, the Sum up to 10 test, and the Visual Patterns Test. In the main part of the study, participants were provided with 16 data visualization types (see above). For all diagrams, participants were asked how familiar they were with this diagram and how good they think they could handle this diagram on a scale from 1 (“Not at all”) to 5 (“Very good”). After that, participants were provided with 4 statements about the data, which they had to judge as either correct or incorrect (including the option to choose “I don't know”). The study lasted about 19 (*SD* = 3,5) min.

#### Design and Data Analysis

Graphical literacy, performance in the visual working memory (VWM) test (number of errors and response time), the performance in the perceptual speed (PS) test (number of errors and response time), and prior experience (education, working in a job where charts and numbers are common and statistical knowledge) served as independent variables in the analyses. The dependent variables comprised the participants' general understanding of the graph (choosing “I don't know” instead of answering the questions), the number of errors made on the tasks with different visualizations, and task solving time. For analyses, an exploratory cluster analysis was conducted, followed by multiple linear regressions (see below for details).

#### Results

**Figure 2:**
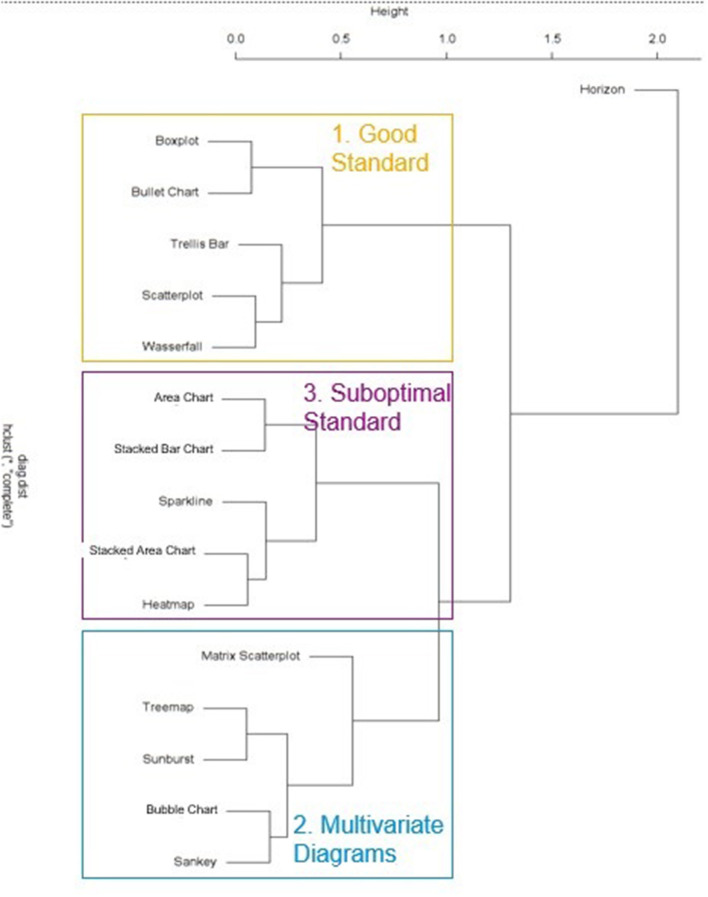
Study 2: Dendrogram of visualization clusters.

**Figure 3:**
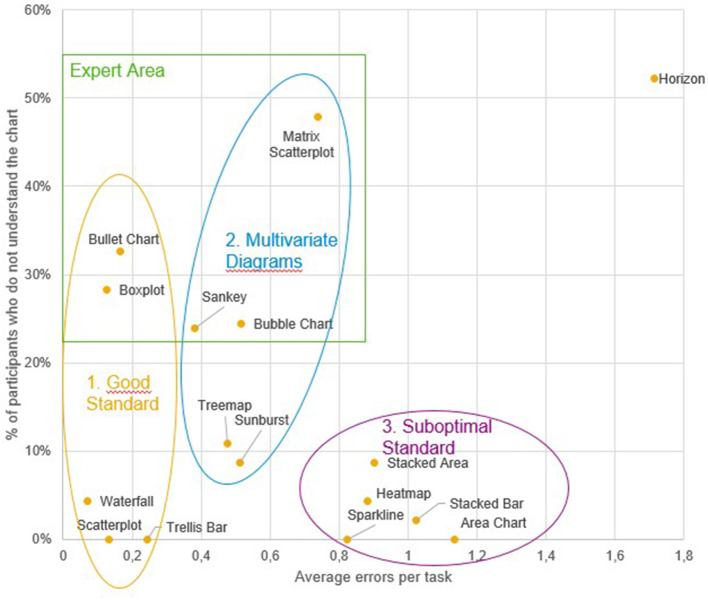
Study 2: Scatterplot with visual representation of the emerging clusters and the artificial expert cluster.

First, to gain an understanding of how the tested visualizations may be related to each other regarding their general susceptibility to errors as well as the self-ratings with respect to the general understanding of these visualizations, a hierarchical cluster analysis was conducted based on the two dependent variables “general understanding” and “number of errors,” which were considered most relevant from an applied perspective. The resulting dendrogram/clusters are shown in Figures 2, 3. Three clusters were derived in total. The first cluster contained error-prone visualizations. The second cluster comprised multivariate and hierarchical visualizations, which were associated with more errors, and which were partially difficult to understand. The third cluster combined all sub-optimal visualizations. These visualizations are common, but do not support error-free interpretation. Horizon charts represented an outlier among all visualizations, as it was both difficult to understand and frequently misinterpreted. Thus, this type of visualization should therefore be generally avoided. The main hypothesis was that some visualizations are more appropriate for participants with substantial prior experience, visual literacy, or cognitive prerequisites necessary to quickly adapt to these visualizations. To address this main hypothesis, all visualization types that were not understood by all participants were grouped into an “expert cluster” (consisting of bullet charts, boxplots, matrix scatterplots, sankeys, and bubble charts). For these visualizations, multiple regression analyses were conducted in order to model the impact of independent user variables on the general understanding, the error rates, and the reaction times as stated in the hypothesis. An overview of results for an initial multiple regression analysis based on general understanding is shown in Table 1. However, note that 18 observations had to be excluded due to missing data on two independent variables (participants who were not active in a job), and the regression model was only marginally significant, *F*_(9,17)_ = 2.48, *p* = 0.051, adj. *R*^*2*^ = 0.339. A second model only considered the factors statistical knowledge and VPT errors (which were the only significant predictors within the full model) and resulted in a significant effect overall, *F*_(2,42)_ = 6.41, *p* < 0.01, adj. *R*^*2*^ = 0.197, including all cases. Finally, as visual working memory is probably difficult to measure in an applied software setting, a third model focusing on statistical knowledge only was calculated. This analysis also resulted in a significant prediction of the general understanding of data visualizations, *F*_(1,43)_ = 6.93*, p* < 0.02, adj. *R*^*2*^ = 0.119. A correlation analysis verified the analysis, as statistical education *r*_(43)_ = −0.37, *p* < 0.02 and visual working memory *r*_(43)_ = 0.36, *p* < 0.02 were significantly correlated with each other. Multiple linear regressions were also run for analyzing error rates, as shown in Table 2. In an initial analysis using all predictors, the factor education was significant, but the whole model was not, *F*_(9,17)_ = 1.04, *p* = 0.450, adj. *R*^*2*^ = 0.013. This model was based on the sample with 19 missing cases on two independent variables (participants who were not active in a job). When excluding the factors responsible for the missing cases (job experience) in a second model, no factor was significant anymore and the model again was not significant, *F*_(7,34)_ = 0.55, *p* = 0.790, adj. *R*^*2*^ = −0.083. Finally, only using the factor education as a predictor also yielded no significant effect, *F*_(1,43)_ = 1.03, *p* = 0.315, adj. *R*^*2*^ = −0.001. The self-reported familiarity with the provided expert data visualization types significantly predicted the number of errors made in the tasks, *F*_(1,43)_ = 4.17, *p* < 0.05, adj. *R*^*2*^ = 0.067, although the effect is not particularly strong.

**Table 1:** Study 2: multiple regression models to predict the general understanding of visualizations in the expert cluster.

**Factor**	**Variable**	***B***	***SE B***	**β**	***t***	***p***
**MODEL 1 (18 MISSING)**						
Graphical literacy	SGL	0.01	0.06	0.03	0.19	0.856
Cognitive variables	VWM error	0.69	0.28	0.48	2.46	0.025^*^
	VWM time	0.01	0.02	0.07	0.36	0.726
	PS errors	−0.22	1.37	−0.03	−0.16	0.875
	PS time	−0.10	1.02	−0.18	−0.01	0.922
Prior experience	Education	−0.25	0.22	−0.23	−1.16	0.261
	Job – Numbers	0.10	0.44	0.05	0.23	0.818
	Job – Graphs	−0.39	0.40	−0.23	−0.98	0.341
	Statistical knowledge	−0.83	0.38	−0.50	−2.16	0.045^*^
**MODEL 2**						
Cognitive variables	VWM error	0.52	0.23	0.31	−2.28	0.028^*^
Prior experience	Statistical knowledge	−0.65	0.27	0.33	−2.42	0.020^*^
**MODEL 3**						
Prior experience	Statistical knowledge	−0.73	0.28	−0.373	−2.632	0.012^*^

* *p < 0.05*.

**Table 2:** Study 2: multiple regression models to predict the errors in the expert cluster.

**Factor**	**Variable**	***B***	***SE B***	**β**	***t***	***p***
**MODEL 1 (18 MISSING)**						
Graphical literacy	SGL	−0.01	0.02	−0.10	−0.46	0.653
Cognitive variables	VWM error	0.03	0.09	0.07	0.31	0.759
	VWM time	0.00	0.01	0.17	0.72	0.481
	PS errors	−0.51	0.42	−0.30	−1.23	0.235
	PS time	−0.51	0.31	−0.38	−1.64	0.119
Prior experience	Education	−0.15	0.06	−0.53	−2.23	0.039 ^*^
	Job – Numbers	0.00	0.13	−0.00	0.00	0.999
	Job – Graphs	0.15	0.12	0.35	1.24	0.233
	Statistical knowledge	0.09	0.16	0.21	0.75	0.464
**MODEL 2**						
Graphical literacy	SGL	−0.02	0.02	−0.16	−0.97	0.339
Cognitive variables	VWM error	−0.00	0.07	−0.01	−0.07	0.946
	VWM time	0.00	0.00	0.01	0.06	0.954
	PS errors	−0.37	0.33	−0.20	−1.15	0.258
	PS time	−0.22	0.22	−0.18	−0.99	0.329
Prior experience	Education	−0.04	0.04	−0.16	−0.93	0.358
	Statistical knowledge	0.02	0.07	0.04	−0.227	0.822
**MODEL 3**						
Prior experience	Education	−0.04	0.04	−0.15	−1.02	0.315

* *p < 0.05*.

#### Discussion

This study identified three clusters of visualization types, namely the “good standard” (Scatterplot, Trellis Chart, Waterfall, Boxplot and Bullet Chart), the “suboptimal standard” (Sparkline, Heatmap, Stacked Bar Chart, Area Chart, Stacked Area Chart), and the “multivariate visualizations” (Sunburst, Treemap, Matrix Scatterplot, Sankey Chart, Bubble Chart). An additional (artificially created) cluster combined Boxplot, Bullet chart, Sankey Chart, Bubble Chart and Matrix Scatterplots into an “expert visualizations” group. Understanding of these visualizations was predicted significantly by the users' statistical knowledge, and therefore this should be considered a crucial factor in providing recommendations for a user. Interestingly, neither the self-reported ability to work with charts nor the SGL score were good predictors for either the probability of understanding a chart or the errors made in the tasks. Also, despite a current debate emphasizing the impact of cognitive variables on learning to work with data visualizations (Velez et al., 2005; Conati and Maclaren, 2008; Toker et al., 2013; Lallé et al., 2015), the effect of perceptual speed on the ability to adapt to unfamiliar data visualizations (or to work more accurately with them) could not be replicated in this study. Visual working memory, on the other hand, was indeed a significant predictor of the ability to understand unfamiliar visualizations (but not of error-free reasoning with them).

#### Limitations

This study also suffered from several limitations. One major limitation was that participants only had to complete a single task based on each visualization type. As the difficulty of the tasks was not controlled independently, the evaluation of visualization types may be quite vulnerable to task-based processing disruptions or task difficulty. Additionally, participants were not a representative sample, as the educational background was nearly exclusively academic. A more diverse sample may yield more nuanced results and provide answers to the question of the extent to which people with lower educational levels can work with different types of visualizations. Furthermore, the online setting of the present study is not a controlled environment and therefore potentially subject to distraction. This may have also influenced the measurement of cognitive abilities, although it may also be argued that in a work setting distractions can actually be considered to occur quite frequently.

### Study 3—Examining the Relationship of User Intents and Low-Level Actions

After examining a selection of user traits in Study 2, the next step was to focus on another aspect closely associated with user traits, namely the more transient user states (e.g., emotions, intentions etc.). Based on the four dissociable user intents monitoring, analyzing, planning, and communicating (assumed to be engaged in a cyclical fashion, see Few, 2004), the two intents monitoring and analyzing were selected for Study 3. We reasoned that these two intents were more closely associated with perceiving and understanding data visualizations, while planning and communicating were rather related to deriving actions. In order to distinguish between analyzing and monitoring, we first focused on low-level task profiles. Specifically, several analysts and managers were interviewed regarding their regular work with data visualizations and the associated tasks. For low-level tasks, we distinguished between the sub-tasks retrieving values, filtering, computing a derived value, finding extrema, sorting, determining ranges, characterizing distributions, finding anomalies, clustering, and correlating (Amar et al., 2005). It was hypothesized that the user intents “analysis” and “monitoring” are associated with significantly different patterns of these low-level sub-tasks. If this holds true, the corresponding visualizations should therefore be different, too.

#### Participants

Fourteen experts (4 female, 10 male) were interviewed. They were recruited via personal network and were questioned via telephone. The participants were from different departments in different companies, ranging from sales management in a small e-commerce startup to controlling in a DAX-30 automotive corporation. All participants had an academic background, and the age range was 25–55.

#### Procedure

After a short introduction and some information regarding the background of the study, all participants were asked to report which data-related tasks (involving visualizations) they were frequently engaged in. One participant could principally report any number of user tasks. After the interviews, each reported user task was assigned to either a monitoring or an analysis intent, and then they were further decomposed into their low-level sub-task components (see above). The reported user tasks were assigned to a monitoring intent if they comprised a check against a point or level of comparison and produced a binary result (e.g., “Control if work hours in every department indicate overtime”). Otherwise, they were assigned to the analyzing intent (e.g., “Checking how much plan and actual were apart in last periods of time”).

#### Results

**Figure 4:**
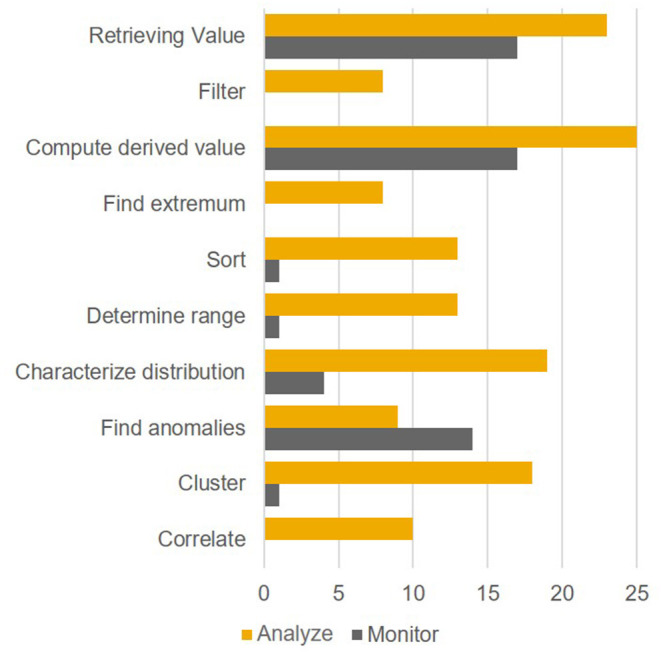
Study 3: Distribution of low-level tasks within the intents.

The 14 participants reported 45 tasks altogether (average 3.2 tasks per participant). Of these 45 tasks, 19 were analysis tasks and 26 were monitoring tasks. By calculating a *t*-test for the mean number of low-level tasks associated with each intent, a significant difference could be observed, *t*_(43)_ = 5.397, *p* < 0.0001. Specifically, tasks associated with an analysis (vs. monitoring) intent involved a significantly greater number of low-level sub-tasks per reported task. A chi-square test also revealed that the distribution of the occurrence of the 10 sub-tasks involved in the two different intent types significantly differed, χ^*2*^ (9) = 42.61, *p* < 0.001. Therefore, our hypothesis was confirmed. The distribution of basic tasks within the two types of intents is illustrated in Figure 4.

#### Discussion

The hypothesis of this study was that the low-level task profiles of monitoring and analysis user states are significantly different. Based on the results of the study this hypothesis can be confirmed, therefore using these intents as a basis for adapting visualizations to user states appears to be reasonable. Because of the high level of relevance for all intents, the retrieval of values as well as computing derived values should in particular be as easy as possible. A major implication of this study was that highlighting anomalies in a monitoring setting is an important feature in data visualizations. Being able to highlight specific aspects, however, implies reserving one (ideally pre-attentively processed) feature specification (e.g., color) for callouts. Following up on this thought, reserving colors for semantic callouts may be considered advisable for a monitoring setup. This would need visualizations to be charted without colors by default.

#### Limitations

This study also had some shortcomings. A major problem was that both the classification of reported user tasks to intents and low-level tasks to reported user tasks was essentially subjective. However, we believe that our criteria were overall quite reasonable, and it was necessary to start at some point. Nevertheless, a more objective classification would be desirable. Also, the sample size was relatively small, and once again only academic participants were assessed. The resulting implications discussed above therefore may thus not be generalizable to user groups with lower education levels.

## Developing a User-Adaptive Visualization Taxonomy

**Figure 5:**
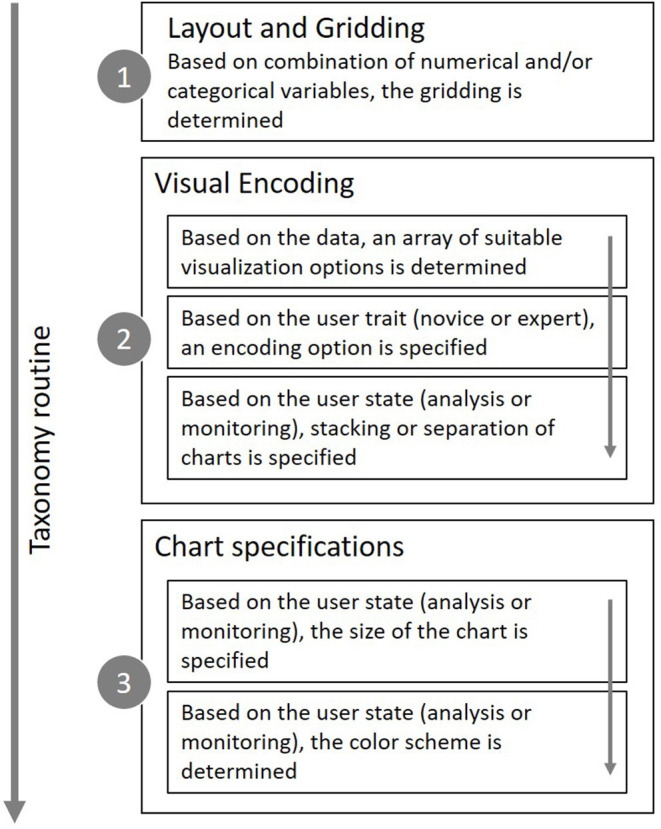
Adaptive taxonomy calculcation steps to determine layout, encoding and specifications.

The main aim of this paper was to provide a first approach for developing systematic user-adaptive visualization recommendations. While previous related studies (see above) can be used as clear guidelines for the general design of data visualizations, the studies described so far represent a reasonable basis for our decision to include adaptive elements based on the user trait “expertise” (Study 2) and the user state “intention” (Study 3). To make the taxonomy applicable for all kinds of data sets, a central requirement was also to provide a structured approach for the visualization of both simple and complex variable settings. The proposed solution, which is mainly grounded on discussions with experts and own prior experience with typical options present in modern data visualization software, is outlined in the following section and summarized in Figure 5.

### Layout and Gridding of Data

**Figure 6:**
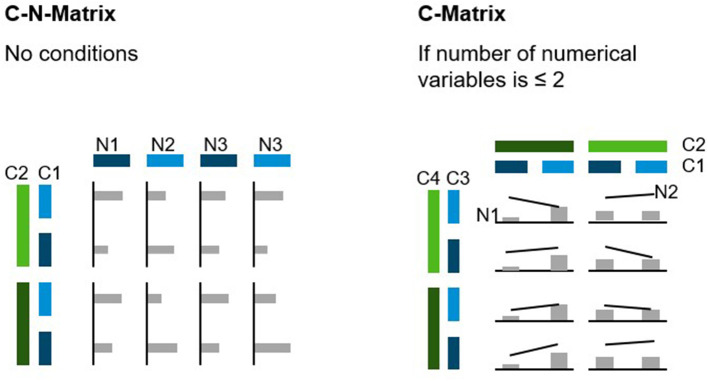
C-N-Matrix and C-Matrix.

If a given data set has more dimensions than can (or should) be displayed in a single chart, a layout of several charts is needed. This grid should combine categorical variables on one axis and numerical dimensions on the other axis (C-N-Matrix). In this way, n-dimensional data sets can be visualized. If there are only up to two numerical variables, these can be displayed directly in the chart, and the second grid axis may also be used for categorical variables (C-Matrix), which is the equivalent of a pivot table. Both the C-N-Matrix and the C-Matrix are displayed in Figure 6. While the C-N-Matrix is the most flexible way to visualize any data set, the C-Matrix may be more space efficient and should therefore be used preferentially.

### Visual Encoding of Queries

Choosing a specific visual encoding type defines which kind of indicators are used to encode the actual data values. For data sets with a 1:1 relationship, bar charts are usually recommended as a visual encoding type in both monitoring and analysis settings due to consistent reports of their superiority over alternative encoding types (e.g., Cleveland and McGill, 1984; Heer and Bostock, 2010; Huestegge and Poetzsch, 2018). However, if one of the variables represent time, a line chart is usually recommended as it promotes the mental processing of developments over time. When a 1:n relationship is present in the data, the default for the monitoring intent should be to aggregate the data (e.g., averaging) and to display it as a bar chart, although there should be an easily accessible control element for switching back to the raw data. Given an analysis intent, a boxplot should be the first choice to display the data distribution in a condensed way, if possible enriched with a violin to account for distribution nuances (Matejka and Fitzmaurice, 2017). If the user lacks sufficient statistical background knowledge or the rendering engine is not able to display this type of graph, the second-best option would be a distribution curve. When distribution visualizations are not available at all, there is no other option than to display the raw data points. For this, strip plots should be preferred over normal dot plots, as the former allow for a much tighter packing of indicators without a substantial risk of “over-plotting.”

It is principally possible to shift one variable from the categorical grid axis to the data encoding region itself through stacking of indicators. The most frequently encountered example for this option is the stacked bar chart. Stacking comes with the benefit of offering the possibility to focus on combined values for comparison purposes (e.g., comparing spending categories across departments). However, beside this distinct benefit stacking may also decrease the speed and accuracy at which a user judges trends within a category, mainly due to the more complicated cognitive demand of aligning and judging indicators without a common baseline (Simkin and Hastie, 1987), as also shown in *Study 2*. Following the general outline of the monitoring intent, which mainly focuses on getting a quick overview over a complex data pattern, a separate display of charts seems to be a reasonable default, while stacking seems to be useful for an analytic intent when the variable at hand represents a sum (not an average, as summing averages is not useful in most contexts).

### Deriving Chart Specification Recommendations

Although the value encoding type is the most prominent feature of any visualization, specifications such as the size of a chart and its coloring can also affect the perception and understanding of charts. Thus, these features should also be considered, in particular as a function of user intent. The optimal chart size in the context of a monitoring intent certainly cannot be determined exactly. However, it should be large enough to allow for an accurate, readable depiction of the visual indicators, but at the same time as small as possible to prevent unnecessary shifts of visual attention (Heer et al., 2009; Heer and Bostock, 2010; Orlov et al., 2016). Such an optimal size should also entail that a numerical scale should roughly fit in the foveal area (5° of the visual field), or at least the parafoveal area (about 8°). Additionally, chart sizing should be flexible enough to account for multiple devices. A useful unit of measurement in this context may be the root em (rem), which is usually considered a standard size in current web design. While desktop setups and devices with lower resolution convert 1 rem to 16 pixels (px) during rendering, high-resolution devices usually transform 1 rem to 32 px (Powers, 2012). This is supposed to ensure optimal readability, as the x-height is above the 0.2° threshold (Legge and Bigelow, 2011). Modeling the optimal rem size for different device scenarios across the visual field (Kaiser, 1996), a sizing of 10 rem has been considered to represent a good choice. If the data are separated, the individual charts can be decreased in size down to around 5 rem, which should still result in accurately readable charts. In the context of an analysis intent, it may be beneficial to provide a larger chart, as this presumably facilitates a more thorough and specific exploration of the data (Orlov et al., 2016). However, the size of the chart should not exceed perifoveal vision, as a significant decrease in stimulu detection occurs beyond 20° of the visual field (see section “Related Previous Works”). The radius of perifoveal vision in the example described above is equivalent to about 333 px. The largest fitting rectangle would be a square with a length of 471 px or 29 rem. Nevertheless, it remains to be considered that the width of the chart depends on the number of data points/categories at hand, and therefore the actual width of a chart may well-exceed the recommended size. For Scatterplots and other encoding types involving numerical variables on both dimensions, these size recommendations apply for both dimensions. Regarding the coloring of charts in a monitoring setting, it may be considered beneficial to refrain from using colors or to restrict coloring to a few desaturated indicator colors (Few, 2009). As a separation of charts is proposed for this type of setting, the colors are likely not needed immediately and can instead be reserved for semantic callouts (e.g., warnings) to increase the visibility of such callouts. For analytic settings, especially those involving stacked variables, more colors may be needed, although desaturated colors may reduce mental distraction in these cases, too.

## Study 4—Validating the Taxonomy With Experts

The taxonomy outlined in the previous section certainly needs empirical validation to prove its usefulness for data visualization recommendation systems. As a first step into this direction, *Study 4* therefore evaluated whether data visualization experts considered the taxonomy-based recommendations for different settings suitable in the context of tasks that closely resemble real-world applications. The hypotheses evaluated in this study were as follows: The visualizations provided by the taxonomy are generally judged as suitable by the experts. Furthermore, we tested whether the taxonomy is suited to visualize even complex data sets without the resulting charts being judged as significantly less suitable than in simple settings.

### Participants

Ten analytics experts from within SAP were interviewed. All participants were male. Seven of the 10 participants held at least a master's degree. The participants reported to frequently work with data, with an average self-rating of 4.4 on a scale from 1 “never” to 5 “very often” (*SD* = 0.84). They judged their statistical education level to be at an average of 3.2 on a scale ranging from 1 to 4 (see Study 1 for details on this scale). The age range was 25–51.

### Stimuli

**Figure 7:**
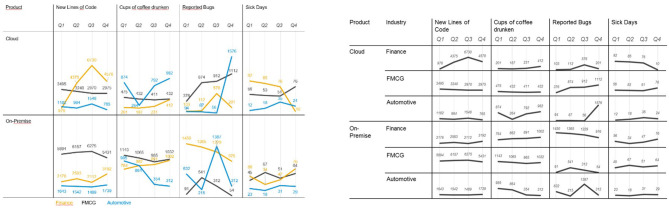
Two visualizations displaying the same data set for an analysis setting **(left)** and a monitoring setting **(right)**.

The participants worked through 12 trials, each consisting of a task and an associated visualization. These 12 trials were built from six data sets, which were each combined with both a monitoring task and an analysis task (in separate trials). The specific visualizations varied across task types as suggested by the taxonomy. The six data sets were characterized by three different degrees of complexities: The easiest settings consisted of three dimensions (one categorical, one numerical, and one time variable), the intermediate settings consisted of five dimensions (two categorical, two numerical, and one time variable), and the most complex settings involved seven dimensions (two categorical, four numerical, and one time variable). Example stimuli are shown in Figure 7.

### Procedure

The experiment was paper-based and completed in presence of an experimenter. After a brief introduction, demographic data were gathered, including age, education, and how much the participants worked with data in their job. Additionally, participants were asked to report their statistical education. In the main part of the experiment, the participants were provided with 12 trials (six monitoring tasks and six analysis tasks). In the monitoring tasks, participants were asked to “mark the data point(s) or dimension(s) you found to stick out and may need deeper analysis.” In the analysis settings, participants were provided with four statements about the data set in a multiple-choice format and asked to mark the correct options. After completing the task in each trial, participants were asked to judge how suitable the given visualization was for the task on a scale from 1 (not suitable) to 5 (very suitable). Each trial ended with an open section for the participants to provide feedback and optimization ideas. All data sets were taken from the field of enterprise performance management, which the participants could relate to. The average study time was 40 min (*SD* = 7.55).

### Design and Data Analysis

Trial setting (analysis/monitoring) and data set complexity (3/5/7 variables) served as independent variables. The dependent variables were the suitability rating scores. For hypothesis testing, a within-subject two-way ANOVA was conducted.

### Results

**Figure 8:**
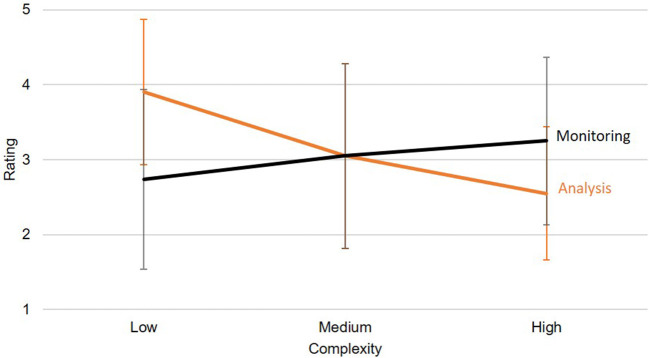
The ANOVA showed a significant interaction effect between the task and the complexity regarding the rating. Error bars show standard deviations.

Results regarding the first hypothesis, namely whether the visualizations are generally judged as suitable by the experts, were rather ambivalent. The average rating amounted to 3.09 (*SD* = 1.17) on a scale from 1 to 5, suggesting that the visualizations proposed based on the taxonomy were rated at an intermediate level of suitability. However, these numbers only reflect one part of the picture: In the verbal feedback for each visualization, it became clear that a lot of factors negatively affected the ratings that were not part of the main study rationale: Unit measures were reported to be unfitting, variable choices were considered invalid regarding their contextual validity, and features such as filtering and aggregating were missed as they were not possible on paper. After the experiment, all participants were familiarized with the idea of an adaptive data visualization taxonomy, and all of them endorsed the idea. Therefore, although the quantitative data are not finally conclusive in this regard, the verbal feedback supported the general usefulness of a taxonomic approach. The second hypothesis proposed that the taxonomy is also useful in visualizing even complex data sets without being judged as significantly less suitable than for simple data sets. To answer this question, a two-way ANOVA was calculated for the experts' ratings. There was no significant main effect for either data complexity, *F*_(2,18)_ = 1.06, *p* = 0.369, nor for task, *F*_(1,9)_ = 0.55, *p* = 0.476. However, we observed a significant interaction, *F*_(2,18)_ = 11.47, *p* < 0.001, η*p*^*2*^ = 0.560. While monitoring settings with higher complexity were rated better than those with lower complexity, this effect was reversed for analysis settings, as shown in Figure 8. It may be argued that in analysis settings, complex data are usually not processed based on a single view, but rather experienced sequentially by interacting with the data using filtering, brushing, aggregating, and drilldown options (which were not available in the present study).

### Discussion

The main aim of this experiment was to provide first evidence for the usefulness of the developed taxonomy for the adaptive display of data. Although it could not be concluded that the specific recommendations derived from the taxonomy were already sufficiently suitable for an instant implementation into existing applications, the general principles of the taxonomy were embraced by our sample of experts. Based on the verbal reports, it appeared that some aspects were already sufficiently useful, for example the scalability in the monitoring mode for scenarios with different complexity. In contrast, the display options for the analytic mode were less well-suited, therefore more work in this regard is necessary.

### Limitations

A main limitation of this study is that user traits were not considered here as the study was conducted in a paper-based manner. Therefore, a fully user-adaptive approach could not be evaluated here. Also, some side effects of our decision to use a paper version negatively affected the suitability ratings (see above). Regarding the stimuli used in this study, it would have been useful to compare different visualizations for each intent/task combination. Through this procedure, it may have become more evident where benefits or disadvantages of specific visualizations are located, and if the taxonomy actually provided good recommendations when compared with other possible solutions. This approach appears to be promising for further research, which should also involve larger sample sizes.

## Conclusion and Future Work

A central claim of the present paper is that data visualizations should be adapted to both the user and the context. This idea was supported by *Study 1*, which demonstrated substantial inter-individual variability among a group of experts when freely choosing an option to visualize data sets. To lay the theoretical groundwork for the envisioned taxonomic approach, a user model combining user traits, states, strategies, and actions was proposed and further evaluated empirically in *Studies 2* and *3*. The results implied that for adapting to user traits, statistical expertise is a relevant dimension that should be considered. Additionally, for adapting to user states different user intentions such as monitoring and analysis should be differentiated and accounted for. These results were used to develop a taxonomy which adapts visualization recommendations to these (and other) factors. For example, a monitoring intention may benefit from separated data lines without coloring, while an analysis intention should benefit from combined charts. In addition to this adaptive approach, the taxonomy also outlined a way to grid up complex data sets to optimize their visualization. A preliminary attempt to validate the taxonomy in *Study 4* tested its visualization recommendations with a group of experts. While the corresponding results were somewhat ambiguous overall, some aspects of the results nevertheless supported the claim that a user-adaptive data visualization approach based on the principles outlined in the taxonomy can be useful. Of course, the present approach to user adaptivity is still quite rudimentary, especially due to the relatively low number of participants. To solidify the results, larger samples should be collected.

In theory, one might want to consider every user to be an individual based on multiple (potentially quantitative) dimensions relevant to visualization adaptivity. Here, we only considered very few of these dimensions, which usually comprised two binary alternatives (e.g., two task-based intents). Thus, more research is needed in order to finally come up with a truly individualized output. Although adaptivity in the context of data visualization is still in its initial stage, it clearly has a lot of potential for future development. The full potential of adaptive visualization will probably be of great relevance especially in more complex settings of decision support involving data visualization, where tailoring information width and depth to the user is mandatory.

One of the areas that need to be worked on more extensively in order to move forward with adaptive visualizations is the contextual component, as “*to create useful adaptive visualization tools we must understand the relationship between a users' context and the visualization they require*” (Oscar et al., 2017, p. 811). Without knowing more about the context, it is only possible to provide a sensible default. One possible option to address this issue could be the use of conversational user interfaces, which would allow the user to articulate context and intentions in more detail and subsequently enable the system to provide more suitable visualizations. Another approach to adaptivity would be to let the system collect data about usage patterns and then to suggest these learned patterns to users later. This approach, also known as collaborative filtering, requires large amounts of data and is essentially theory-blind: While it does not need any theoretical assumptions in order to work, it cannot take into account basic knowledge about which behavioral, user- or context-related aspects can be meaningfully combined. Due to this serious problem, it may be concluded that knowledge-based filtering may represent a reasonable middle way (Vartak et al., 2017).

Another area crucial to the implementation of such a taxonomy the issue of standardization. This is relevant for both the design of charts as well as for how visualizations are coded. For example, chart rendering engines are usually not compatible to each other. A single system is not able to control different engines as their required input format differs, although first steps toward a standardized encoding format have been taken with the introduction of *CompassQL* (Wongsuphasawat et al., 2016). Nevertheless, the corresponding problems do not only affect rendering engines: A micro-service-based approach to the whole data visualization ecosystem could also encompass data query recommendation or statistical modules. This would provide a truly flexible system that could not only adapt visualizations, but also adapt the information that is displayed and how it is further computed. This would clearly be a desirable development in the future.

When considering the rising complexity of data and information in the world, it appears evident that even adapted data visualization cannot be the sole solution to making this information truly accessible for users. Adaptivity may ultimately be understood as providing an individualized information display and decision support. To enable this, a shared semantics between users and systems needs to be developed. Only through teaching the machine how virtual (data) objects relate to each other, the system may be able to provide useful decision support that not only follows a comprehensible logic, but also considers the individual users as cognitive beings that are also prone to typical judgement (and other cognitive) biases. This next step toward individually aiding users in their data-driven decisions can also be considered a step toward artificial intelligence, as we enable machines to apply human (user-centered) perspectives.

## Data Availability Statement

All datasets generated for this study are included in the article/Supplementary Material.

## Ethics Statement

Ethical review and approval was not required for the study on human participants in accordance with the local legislation and institutional requirements. The patients/participants provided their written informed consent to participate in this study.

## Author Contributions

The paper was outlined and conceived by TP. All authors contributed to the design of the study. TP organized the dataset, performed the statistical analyses, and wrote the first draft of the manuscript. All authors contributed to manuscript revision, read and approved the submitted version.

### Conflict of Interest

TP and PG were employed by the SAP SE. The remaining author declares that the research was conducted in the absence of any commercial or financial relationships that could be construed as a potential conflict of interest. The authors declare that this study received no further funding by SAP SE. SAP SE was not involved in the study design, collection, analysis, interpretation of data, the writing of this article or the decision to submit it for publication.

## Supplementary Material

Datasheet S1 Study 1 raw data.

Datasheet S2 Study 2 raw data.

Datasheet S3 Study 3 raw data.

Datasheet S4 Study 4 raw data.
